# A consensus statement on minimum operational standards for geriatric emergency care in Belgium: a modified Delphi study

**DOI:** 10.1186/s12877-023-04474-0

**Published:** 2023-11-22

**Authors:** Pieter Heeren, Farah Islam, Didier Desruelles, Johan Flamaing, Marc Sabbe, Koen Milisen, the Belgian URGENT Delphi Group, Philippe Heerinckx, Ives Hubloue, Tuan Long Tran, Stefan Wynants, Veronique Ghekière, Isabelle De Brauwer, Laetitia Beernaert, Sophie Cristelbach, Sven Guldemont, Dieter Lumen, Sebastien Sohet, Emilie Bogaerts, Nadja Himschoot, Nicole Michaux, Mayelise Dath, Robin Biets, Cecile Piron, Koen Van den Heede, Carine Vande Voorde, Celine Ricour

**Affiliations:** 1 Department of Public Health and Primary Care, Academic Centre for Nursing and Midwifery, KU Leuven, Kapucijnenvoer 35/4, Leuven, 3000 Belgium; 2 Department of Geriatric Medicine, University Hospitals Leuven, Herestraat 49, 3000 Leuven, Belgium; 3 Research Foundation Flanders, Egmontstraat 5, 1000 Brussels, Belgium; 4 Department of Public Health, Institute of Nursing Science, University of Basel, Bernoullistrasse 38, 4056 Basel, Switzerland; 5 Department of Emergency Medicine, University Hospitals Leuven, Herestraat 49, 3000 Leuven, Belgium; 6 Department of Public Health and Primary Care, Emergency Medicine, KU Leuven, Kapucijnenvoer 35/4, 3000 Leuven, Belgium; 7 Department of Public Health and Primary Care, Gerontology and Geriatrics, KU Leuven, Herestraat 49, 3000 Leuven, Belgium

**Keywords:** Emergency department, Observation unit, Older adults, Geriatric emergency medicine

## Abstract

**Background:**

As emergency department (ED) leaders started integrating geriatric emergency guidelines on a facultative basis, important variations have emerged between EDs in care for older patients. The aim of this study was to establish a consensus on minimum operational standards for Geriatric ED care in Belgium.

**Methods:**

A two-stage modified Delphi study was conducted. Twenty panellists were recruited from Dutch and French speaking regions in Belgium to join an interdisciplinary expert panel. In the first stage, an online survey was conducted to identify and define all possible elements of geriatric emergency care. In the second stage, an online survey and online expert panel meeting were organized consecutively to determine which elements should be recognized as minimum operational standards.

**Results:**

Between March 2020 and February 2021, the expert panel developed a broad consensus including ten statements focusing on the target population, specific goals, availability of geriatric practitioners and quality assurance. Additionally, the expert panel also determined which protocols, materials and accommodation criteria should be available in conventional EDs (39 standards) and in observational EDs (57 standards).

**Conclusions:**

This study presents a consensus on minimum operational standards for geriatric emergency care in two ED types: the conventional ED and the observational ED. These findings may serve as a starting point towards broadly supported minimum standards of care stipulated by legislation in Belgium or other countries.

**Supplementary Information:**

The online version contains supplementary material available at 10.1186/s12877-023-04474-0.

## Background

The traditional fast-paced and problem-focused emergency department (ED) model does not enable optimal care delivery to the growing group of older adults (i.e. persons over 65 years of age) [1, 2]. This is illustrated by several studies reporting that problems specific to these segments of the population (e.g. cognitive impairment, falls) are often missed during ED disposition planning [3]. Older adults are therefore frequently (in comparison to their younger counterparts) at increased risks for adverse events, such as death, unplanned readmission and functional decline [4, 5].

Despite a clear need for Geriatric ED care, convincing policymakers to invest in this new branch of Emergency Medicine remains a global challenge. The main reason for this is that to date, geriatric emergency care models have not yet been shown to be compellingly cost-effective [6–9]. However, as these findings are affected by methodological limitations, it seems unethical to withhold older adults from safe and low complex care improvements, such as delirium and fall prevention, until its evidence is sufficiently robust [10]. Therefore, several ED leaders started operationalising geriatric emergency guidelines [11, 12]. While some EDs already managed to achieve comprehensive, high-quality standards in this field, others are focused less on this topic [13, 14]. Consequently, important between-ED variations in care for older adults have emerged.

In Belgium, federal legislations have been established to guide minimum operational standards for in-hospital care. For example, there is federal legislation describing the minimum standards for the hospital-based care programme for geriatric patients [15]. Additionally, there is also federal legislation organizing the function ‘specialised emergency care’ [16]. However, a specific focus on geriatric care in the ED is lacking in both legislations. As the ED is the gateway to in-hospital care for many vulnerable older adults and because the need for Geriatric ED care is well-recognized across healthcare workers in Belgian EDs, we therefore argue that as a next step, it is important to work towards the implementation of national or system-specific geriatric emergency care standards [17]. As a first step towards this goal, this study aims to establish clinical consensus on minimum operational standards that should be required for Geriatric ED care settings in Belgium.

## Methods

### Study design

A two stage modified Delphi study was conducted based on Veugelers’ guide for design choices in Delphi studies [18, 19]. The first stage aimed, via an online survey, to identify and define all possible elements of geriatric emergency care. The second stage aimed to classify level of importance of various geriatric emergency care elements, which was obtained via an online survey and online consensus meeting.

### Expert panel

High quality care for older adults requires collaborative efforts between multidisciplinary healthcare professionals [20]. As such, we aimed to create an expert panel which included ED physicians, ED nurses, geriatricians, geriatric nurses working in inpatient geriatric consultation teams and healthcare policy experts (four experts in each category for a total of 20 panellists). Furthermore, as Belgium is comprised of separate Dutch and French speaking regions, we also aimed to include an equal number of experts from each of the regions. All panellists who were invited to participate in our research were experts with scientific/professional interests in improving geriatric emergency care. All invitations were sent by e-mail.

### Decision-making rules

Several pre-defined decision-making rules were applied in our research to process the input of expert panel members. First, consensus was achieved when at least 70% of the expert panel members agreed or disagreed on every aspect of a question. Second, comments or requests to adjust an element definition or add answer categories were brought to the expert panel only if at least two panellists had a similar comment or request. The relevance and value of a comment or request addressed by only one panellist were discussed during internal research team meetings to evaluate whether it should be further evaluated by the panellists or not.

### Stage 1: identifying and defining all possible geriatric emergency care elements

Stage 1 of this study included one part. Based on the core documents in geriatric emergency medicine, such as the Silver book [12], the American Geriatric ED guidelines [11], the American Geriatric ED accreditation framework [21] and the McCusker framework [22], PH drafted an initial list of geriatric emergency care elements and their definitions in Dutch. This list was evaluated and revised during two research team meetings and, finally, converted into a survey, in which the following question was asked for each care element (e.g. a delirium screening protocol): “Do you agree that this element (and its definition) is a component of geriatric emergency care?” If the respondent answered yes, no additional questions were asked. If the respondent answered no, an open text field was prompted for further suggestions. Additionally, expert panel members could also propose to add or delete elements. The final version of the Dutch survey was translated into French. Depending on the expert panel member’s working language, the respondent completed the Dutch or French version of the survey via ‘Qualtrics’ software [23]. All results were analysed by PH and further discussed during research team meetings.

### Stage 2: Prioritizing geriatric emergency care elements for the Belgian context

Stage 2 included two consecutive parts. In the first part, panellists were asked to rate the necessity of geriatric emergency care elements within two different ED models of care. These were the ‘conventional ED’ and the ‘observational ED’, which have an intended length of stay of four and 24 h, respectively. The definition of these care models is described in the results section. The necessity of each item in both care models was measured using a 9-point Likert scale, ranging from 1 (clearly not necessary) to 9 (clearly necessary) [24]. Respondents knew before completing the survey that their Likert scale scores would be transformed into three groups (based on the MoSCoW-method [25]). Group one was called a ‘must have or minimum standard’ (scores 7–9: without this element, it is not feasible to deliver adequate emergency care for older patients). Group two was called a ‘should have’ (scores 4–6; this element is very desirable, but without its availability, it is feasible to deliver adequate emergency care for older patients). Group three was called a ‘could have’ (scores 1–3; this item will only be available in case of sufficient time and resources). Next, expert panel members rated the extent to which they agreed (i.e. not at all/partly/completely) on statements concerning organisational aspects, such as the target population or how geriatric emergency care goals differ from regular ED care goals. If a panellist did not (completely) agree with a statement, an open text field was prompted for further suggestions. Responses were analysed and discussed as reported in stage 1.

In the second part of this stage, a final online consensus meeting was organized where the findings of part 1 were presented and discussed until consensus was reached according the decision making rules described above.

## Results

### Expert panel

All except two invited experts accepted the invitation to participate in this study. One expert decided not to participate due to no time. The other passed the invitation to a colleague, who accepted.

The final expert panel included three ED physicians, five geriatricians, four ED nurses, three health care policy experts and five geriatric nurses working in an inpatient geriatric consultation team (*n* = 20). This panel was well balanced and diverse for several reasons. For example, expert panel members had on average 19 years of relevant professional experience (minimum–maximum: 2–33 years) and six experts were appointed to management positions (i.e. 2 heads of departments, 4 head nurses). In total, eight experts were affiliated with Flemish hospitals (i.e. Dutch speaking and in the northern part of Belgium) and nine with Walloon hospitals (i.e. French speaking and in the southern part of Belgium). Three experts were working for a bilingual health care policy organisation. Six experts were affiliated with a university hospital.

### Research process

**Fig. 1:**
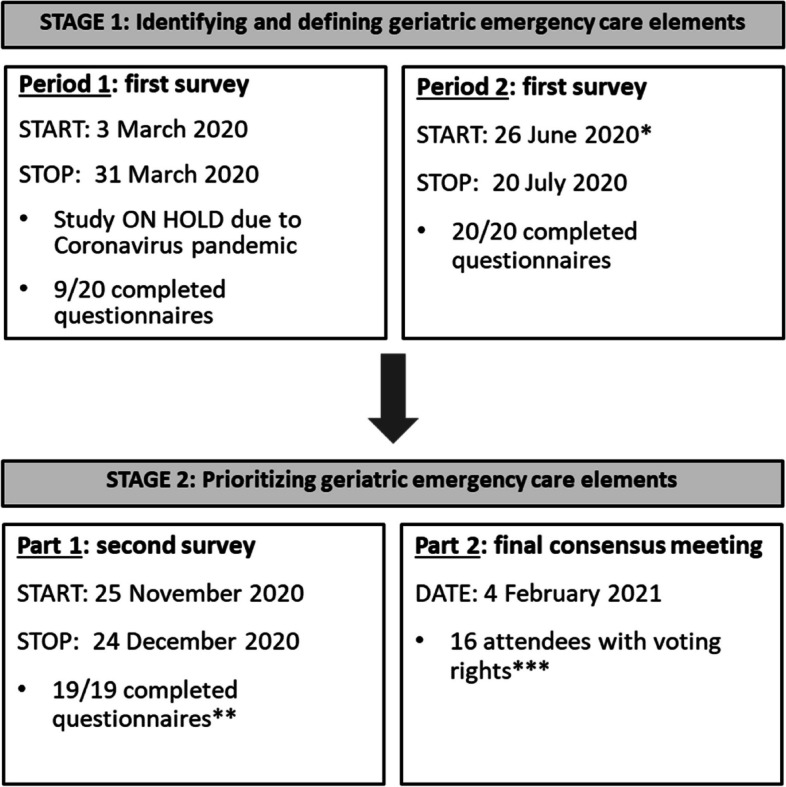
Research process *Expert panel members were invited to complete the survey or adapt their responses, as the experience of the Coronavirus pandemic might have led to new insights and opinions. **One expert panel member decided not to complete the survey due to lack of clinical expertise. ***Thirteen expert panel members and five members of the research team (i.e. PH, FI, DD, MS and KM) attended the consensus meeting. All attendees except the chairperson (PH) and reporter (FI) had voting rights during the meeting (*n* = 16; 5 geriatricians, 4 emergency physicians, 4 geriatric nurses, 2 emergency nurses, 1 health care policy expert. One expert panel member could not attend the entire meeting)

Figure 1 describes the details of the data collection process. In stage 1, a consensus was found for 41 out of 50 proposed geriatric emergency care elements and their definitions. In the first part of stage 2, 29 elements were determined as minimum geriatric care standards in conventional EDs. For observational EDs, panellists established 18 additional standards (Supplementary Tables 1, 2 and 3). Furthermore, consensus was also found for three statements describing organisational aspects (Table 1). After the consensus meeting, the minimum operational standards included ten statements (Table 1) with 39 and 57 geriatric emergency care elements for conventional and observational EDs, respectively (Supplementary Tables 1, 2 and 3).

**Table 1:** Minimum operational standards for geriatric emergency care in Belgium

Article	P1	P2	Remark
*Article 1 – Target group*	**-**	15/16	/
*Article 2—Objectives*	17/19	16/16	Although this was a consensus-achieved item in part 1 of phase 2 (i.e. survey), it was discussed during part 2 of phase 2 (i.e. meeting)
*Article 3 – Conventional ED*	-	14/16	/
*Article 4 – Observational ED*	-	13/16	/
*Article 5 – Summary list*	-	-	There was no formal voting on this article, as it summarizes specific action points for high quality geriatric emergency care (elaborated in Supplementary Tables 1–3 and article 6–10)
*Article 6 – Availability geriatrician and/or IGCT member in conventional ED*	-	15/15	/
*Article 7—Availability geriatrician and/or IGCT member in observational ED*	-	15/15	/
*Article 8 – Training and equipment*	18/19	14/15	Although this was a consensus-achieved item in part 1 of phase 2 (i.e. survey), it was discussed during part 2 of phase 2 (i.e. meeting)
*Article 9 – Coordination and organisation*	15/19	14/15	Although this was a consensus-achieved item in part 1 of phase 2 (i.e. survey), it was discussed during part 2 of phase 2 (i.e. meeting)
*Article 10 – Functional partnership*	**-**	14/15	/

P1 = The number of expert panel members that indicated during part 1 (of stage 2) that the element considered should be a minimum standard; P2 = The number of expert panel members that indicated during part 2 (of stage 2) that the element considered should be a minimum standard

*ED* emergency department, *i.e.* id est, *IGCT* inpatient geriatric consultation team

### Minimum operational standards for geriatric emergency care in Belgium (Table 1)

Art 1. The minimum standards for geriatric emergency care are targeted at:

- the population of older adults, as defined in the Belgian federal legislation on the hospital-based care programme for geriatric patients (Supplementary Table 4)
- the population of (younger) adults with a profile similar to that of the geriatric patient in the Belgian federal legislation on the hospital-based care programme for geriatric patients. (This includes patients whose chronological age is considered too low for formal geriatric referral, but who might benefit from geriatric care principles. For example, a frail 63 year old patient with chronic obstructive pulmonary disease, combined heart-renal failure, depressive mood, memory problems, functional limitations and fall risk).
- These populations will be described as the target group in the following articles.

Art 2. The objective of the minimum standards for geriatric emergency care is threefold and aims to:

1. Assess the risk of atypical presentation of a serious condition.
2. Orient the patient to the most appropriate location in the care system.
3. Ensure continuity of geriatric care aspects.

Art 3. All EDs must meet at least the minimum standards of geriatric emergency care for conventional EDs. These are EDs with an intended length of stay of no more than four hours and where the main objective is, after physical triage, to obtain a differential diagnosis (mainly distinguishing between urgent and less urgent conditions), to start essential treatment and refer the patient.

Art 4. The minimum standards of geriatric emergency care for conventional EDs are extended with additional criteria if the ED is equipped with observation facilities for the target group. This organisational model will hereinafter be defined as an ‘ED with geriatric-focused observation beds’ or an ‘observational ED’. An observation stay extends the intended ED length of stay to a maximum of 24 h and combines the objectives of a conventional ED with the intention of avoiding unnecessary hospitalisation (and the associated risks and costs). The observation period is used for patients who presumably do not require hospitalisation, but need to stay longer in the ED for reassessment or to conduct additional testing, treatments and/or consultations [26, 27].

Art 5. The minimum standards of geriatric emergency care are guaranteed by the use of:

1. Specific clinical protocols and guidelines (Supplementary Table 1)
2. Specific materials and equipment (Supplementary Table 2)
3. Specific accommodation criteria (Supplementary Table 3)
4. Availability of a geriatrician and/or a member of the inpatient geriatric consultation team
5. Quality control.

Art 6. Conventional EDs must ensure availability of a geriatrician and/or a member of the inpatient geriatric consultation team within the locoregional hospital network, according to predefined arrangements. This person must at least be available by telephone for advice during daytime hours on weekdays and weekends.

Art 7. EDs with geriatric-focused observation beds should be able during daytime hours on weekdays and weekends to call on a geriatrician and/or a member of the inpatient geriatric consultation team to establish a geriatric treatment plan and coordinate its implementation in consultation with the locoregional primary care network.

Art 8. The chief medical officer, the head of the ED and the head of the geriatric care programme must ensure that ED staff, geriatricians and members of the inpatient geriatric consultation team are sufficiently trained and equipped to guarantee the minimum standards of geriatric emergency care.

Art 9. The coordination and organisation of the minimum standards for geriatric emergency care are the responsibility of the head of the ED in consultation with the head of the care programme for the geriatric patient.

Art 10. If an ED is part of a hospital without a care programme for the geriatric patient, this ED must set up a functional collaboration with a hospital within the locoregional hospital network that does have a care programme for the geriatric patient.

## Discussion

There is an international push for EDs to adapt their structure and care processes to the complex needs of older adults [6]. As these initiatives introduced variations in care for older adults between EDs, system-specific minimum standards for Geriatric ED care are necessary. As a first step towards initiating national or system-specific operational standards, this study aimed to establish consensus on minimum operational standards required for Geriatric ED care in Belgium.

The consensus obtained in our research was very broad and aligned largely with the high quality level defined by the American Geriatric ED Accreditation (GEDA) Program [11, 21]. The main difference between the GEDA Program and the current consensus is that the extent of standards in the current consensus is determined by ED type, namely a conventional ED and an observational ED. The conventional ED represents the basic ED fulfilling the absolute minimum standards of emergency care in general and for older adults. Observational EDs should be considered the ED type for (more) comprehensive and advanced geriatric care as they have greater availability of a geriatric practitioner as well as geriatric(-friendly) protocols, equipment and accommodation criteria. Another important difference concerns the possibility to determine the extent of Geriatric ED care quality, which is considered by the GEDA Program (e.g. availability of three quality levels) but impossible to consider in the current consensus due to the fundamental differences between accreditation initiatives and minimum requirements imposed by legislation.

Despite the differences between the methods (e.g. accreditation, minimum standards set by legislation) available to achieve high quality geriatric emergency care on a health system level, the pathways towards achieving this goal are similar as they both require a step-by-step effort to be made over the course of several years. As a first step towards this process, it is advisable that EDs (preferably in collaboration with professional organisations) first prioritize changes which are easily accessible and feasible to attain, such as the acquisition and correct use of recommended materials (e.g. walking aids). This aligns with the findings of Kennedy and colleagues describing that the rapid growth of EDs in the United States receiving GEDA is mainly driven by Level 3 accreditation [28]. This is the lowest GEDA level, which is very accessible and feasible for EDs aiming to start improving geriatric emergency care. However, by integrating health system-wide legislation determining geriatric emergency care standards, there is greater potential for policy and government representatives to push the boundaries of goals, which are hard to achieve for individual EDs, such as development of performant transmural communication platforms and stimulating partnerships between care organisations towards collaborative networks. In addition, system-wide uniformity in geriatric emergency care will ensure the facilitation of integrating specific guidelines or protocols into the basic training of ED staff. These examples make it clear that organizing efficient geriatric emergency care requires efforts both within and outside the ED setting.

This study has several strengths and limitations. First, although the expert panel was instructed to reflect on improvement opportunities for the Belgian context, the findings of this study seem generalizable to other countries, as well. We state this because the results of this Delphi study describe minimum quality standards in two general organisational models whose principles are already being deployed in multiple countries. For example, a scoping review on the structure and processes of emergency observation units with a geriatric focus described that geriatric-focused observation beds are already available in emergency care settings within the United States of America, Denmark, United Kingdom, Australia, Singapore, Hongkong and Switzerland [27]. Second, the predefined level for consensus (i.e. agreement among at least 70% of expert panel members) may have influenced the findings of this study. Although there is no universal guideline for the recommended level of consensus, commonly applied levels vary between 51 and 80% [18, 19]. The 70% consensus level was also applied in an earlier Delphi study in the field of Emergency Medicine [19]. Applying a stricter consensus level post factum (e.g. agreement among at least 80% of panellists) could make the consensus less extensive and might be useful to better identify broadly supported elements. However, this exercise also yields the risk of excluding important items. For example, in part 1 of stage 2, there was a 70% consensus for a fall and fracture prevention protocol, but for none of the walking aids. Following discussion during the expert panel meeting about why walking aids are valuable in the ED (i.e. to prevent falls during ED stay, avoid unnecessary admissions and send a patient home safely), the expert panel agreed that the availability of walking aids should be a minimum standard. Furthermore, three types of walking aids reached a consensus level of 80% during the meeting, while the consensus level related to a fall and fracture prevention protocol remained unchanged (i.e. 70%), as it was not revoted during the meeting. Third, one research team member and six out of 19 experts could not attend the expert panel meeting, which might have influenced study findings (see legend of Fig. 1 for more details). Nevertheless, it is noteworthy that the consensus reached during this meeting was generally high (i.e. 80% level). It is also important to note that an external board did not review the study findings. As this is recommended before integrating Delphi study findings into practice, this is not a genuine limitation because the current study was intended to create debate [29].

Further research will be necessary to implement geriatric emergency care in Belgium. Research advancements should focus on refinement of the current consensus and its protocols (e.g. delirium screening and management). This also includes exploring implementation aspects (e.g. acceptability, feasibility, barriers, facilitators) of protocols in multicentre studies and the development of indicators to monitor and benchmark process variables and outcomes [30, 31]. Indicator development was beyond the scope of this research aim but its importance was nevertheless expressed indirectly through various statements on quality assurance by the experts (i.e. art. 8–10).

## Conclusions

This study presents a broad and interdisciplinary consensus on minimum operational standards for geriatric emergency care in two different ED types: the ‘conventional ED’ and the ‘observational ED’. Albeit a first step, these findings will ultimately serve as an important stepping stone for clinical leaders and policy makers aiming to initiate or expand geriatric emergency care initiatives. Although the current consensus was developed with regards to the perspective of the Belgian healthcare system, its results are also applicable to other healthcare systems.

### Supplementary Information

**Additional file 1: Supplementary Table 1.** Specific clinical protocols and guidelines within the minimum standards for geriatric emergency care in Belgium. **Supplementary Table 2.** Specific materials and equipment within the minimum standards for geriatric emergency care in Belgium. **Supplementary Table 3.** Specific accommodation criteria within the minimum standards for geriatric emergency care in Belgium. **Supplementary Table 4.** Article 3 of the Belgian federal legislation on the hospital-based care programme for geriatric patients.

## Data Availability

All data generated or analysed during this study are included in this article and its supplementary files.

## Abbreviations

ED: Emergency department
e.g.: Example given
GEDA: Geriatric ED Accreditation
i.e.: Id est
