# Supplementary material for: A consensus statement on minimum operational standards for geriatric emergency care in Belgium: a modified Delphi study
Source: BMC Geriatr. 2023 Nov 22;23:768. doi: 10.1186/s12877-023-04474-0 (PMC10666396; doi:10.1186/s12877-023-04474-0)
Supplement: Supplementary file 1 — Additional file 1: Supplementary Table 1. Specific clinical protocols and guidelines within the minimum standards for geriatric emergency care in Belgium. Supplementary Table 2. Specific materials and equipment within the minimum standards for geriatric emergency care in Belgium. Supplementary Table 3. Specific accommodation criteria within the minimum standards for geriatric emergency care in Belgium. Supplementary Table 4. Article 3 of the Belgian federal legislation on the hospital-based care programme for geriatric patients. [file 12877_2023_4474_MOESM1_ESM.docx]

**Supplementary table 1. Specific clinical protocols and guidelines within the minimum standards for geriatric emergency care in Belgium**

| **Protocol/Guideline** | **P1** | **P2** | **Consensus definition** |
| --- | --- | --- | --- |
| *Medical referral data* | 18/19 | - | A protocol, established by general practitioners, the ED and the geriatric department, to determine which patient-related data must be provided when an older adult is referred to the ED. (Sharing of these data should preferably take place electronically). |
| *Nursing referral data* | 17/19 | - | A protocol, established by community care nurses, residential care facilities, the geriatric department and the ED, to determine which patient-related data must be provided when an older adult is referred to the ED. (Sharing of these data should preferably take place electronically). |
| *Prehospital care* | 16/19 | - | A protocol, established by general practitioner organisations, the ED and the geriatric department to focus on geriatric care aspects in prehospital settings. For example: if possible, surveying important geriatric problems of the patient (such as a recent fall, attention function, memory...) and ensuring that all important items related to the patient, such as home medication schedule, glasses and hearing aids are brought to the ED. |
| *Triage at the ED* | 17/19 | - | A short protocol established by the ED and the geriatric department to allow the triage team to assess the risk of atypical presentations of serious conditions in patients. |
| *Pain* | 18/19 | - | A protocol established by the ED and the geriatric department enabling ED physicians and nurses to prevent, recognise and treat pain in older patients. |
| *Delirium* | 17/19 | - | A protocol established by the ED, the (geriatric) psychiatry department and the geriatric department for prevention, early recognition and treatment of delirium in patients. |
| *Restraint measures* | 16/19 | - | A protocol established by the ED and the geriatric department to minimise the use of restraint measures in patients. |
| *Fall and fracture prevention* | 14/19 | - | A protocol established by the ED and the geriatric department for primary and secondary fall and fracture prevention in patients. This protocol should also include different referral options. |
| *Medication reconciliation* | - | 14/14 | The report of the attending physician should describe that the current drug therapy was evaluated with particular attention to possible adverse drug events in patients. |
| *Medication rationalisation* | - | 13/13 | A protocol established by the ED, the geriatric department, the clinical pharmacy department and the locoregional general practitioners organisation(s) to minimise the use of unnecessary and/or potentially harmful drugs in patients who are not hospitalised. This is part of basic inhospital care in patients who are hospitalised. |
| *Elder abuse* | 14/19 | - | A protocol established by the ED and the geriatric department for the identification and management of elder abuse in patients (e.g. physical, psychological, financial, sexual and neglect). |
| *Palliative and terminal care* | 15/19 | - | A protocol established by the ED and the geriatric department enabling ED physicians and nurses to inform and support the patient (and their family) in their decision on therapeutic options and, if necessary, the initiation of palliative or terminal care. |
| *Geriatric follow-up* | - | 14/15 | Within the topic of ‘geriatric follow-up’, two protocols are proposed in function of the patient's discharge destination:   1. A protocol established by the ED and the geriatric department to provide a geriatric follow-up by the inpatient geriatric consultation team when necessary. 2. A protocol established by the ED, the geriatric department and the locoregional general practitioner organisation(s) to provide a geriatric follow-up at the geriatric day-hospital or geriatric consultation when necessary. |
| *Discharge instructions for patients and informal caregivers* | 14/19 | - | A protocol established by the ED and the geriatric department on instructions for how to adapt and clearly communicate discharge-related information with the patient and their (informal) caregiver. (e.g. large font, lay language, re-summarizing what has been discussed with the patient). |
| *Discharge instructions for community nurses and nurses working in residential care facilities* | 17/19 | - | A protocol to provide community nurses and/or nurses from residential facilities with patient-related data that are essential at the time of patient discharge and are necessary to optimise continuity of care. |
| *Functional screening** | 15/19 | - | A post-triage screening protocol established by the ED and the geriatric department enabling ED physicians and nurses to detect functional decline in the patient. This protocol should also include different referral options. |
| *Cognitive screening** | 14/19 | - | A post-triage screening protocol established by the ED, the (geriatric) psychiatry department and the geriatric department enabling ED physicians and nurses to detect and register cognitive problems in patients. This protocol should also include different referral options for cognitive problems. |
| *Behavioural and psychological symptoms of dementia** | 15/19 | - | A protocol established by the ED, the (geriatric) psychiatry department and the geriatric department for prevention and treatment of behavioural and psychological problems as part of dementia/neurocognitive diseases. This protocol should also include different referral options. |
| *Substance abuse** | 14/19 | - | A protocol established by the ED, the (geriatric) psychiatry department and the geriatric department for the detection and management of problems related to substance abuse (such as alcohol, medications and/or drugs). This protocol should also include different referral options. |
| *Skin problem** | 14/19 | - | A protocol established by the ED and the geriatric department for ED doctors and nurses to prevent and initially treat skin problems in patients (e.g. such as pressure ulcers, moisture lesions, dermatitis). |
| *Food and drinks** | 15/19 | - | A protocol established by the ED and the geriatric department for ED doctors and nurses to offer patients food and drinks as appropriate to their needs and to minimise the ‘nihil per os’ status (both for diagnostic/therapeutic reasons and when swallowing problems are presumed). |
| *Disposition planning** | 17/19 | - | A protocol, established by general practitioner organisations, community nurses organisations, the ED and the geriatric department to facilitate safe discharge planning of patients with attention for continuity of care and early involvement of informal (e.g. family) and formal caregivers in primary care. |
| *Transfer to residential care facility** | 15/19 | - | A protocol to facilitate patient transfers to a residential care facility. |

ED = emergency department, e.g. = example given.

*This element is not a minimum standard for conventional EDs, but is for **EDs with geriatric-focused observation beds**.

P1 = The number of expert panel members that indicated during part 1 (of stage 2) that the element considered should be a minimum standard; P2 = The number of expert panel members that indicated during part 2 (of stage 2) that the element considered should be a minimum standard.

**Supplementary table 2. Specific materials and equipment within the minimum standards for geriatric emergency care in Belgium**

| **Materials and equipment for conventional EDs** | **P1** | **P2** |
| --- | --- | --- |
| Wheelchairs | 17/19 | - |
| High-low beds/ stretchers | 17/19 | - |
| Pressure-reducing seat cushions and mattresses | 14/19 | - |
| Materials to warm up a hypothermic patient | 18/19 | - |
| Bladder scan | 16/19 | - |
| Urinal | 19/19 | - |
| Toilet chair | 18/19 | - |
| Pictograms overview (in case of language barrier) | 15/19 | - |
| Cane | - | 11/12 |
| Four-wheeled walker | - | 10/12 |
| Two-wheeled walker | - | 9/12 |
| Walking frame | - | 10/12 |
| Non-slip socks | - | 10/12 |
| Lumbar belt and wrist and ankle straps | - | 11/12 |
| Front tray/table that can be fixed to a recliner chair | - | 10/12 |
| Toilet seat raiser* | 14/19 | - |
| Condom catheters* | 14/19 | - |
| Height-adjustable bedside table with lockable wheels* | 18/19 | - |
| Positioning cushions* | 16/19 | - |
| Patient lifting device* | 16/19 | - |
| Patient transfer board* | 14/19 | - |
| Adapted eating and drinking material (such as anti-tremor cup, adapted cutlery...)* | 15/19 | - |

*This element is not a minimum standard for conventional EDs, but is for **EDs with geriatric-focused observation beds**.

P1 = The number of expert panel members that indicated during part 1 (of stage 2) that the element considered should be a minimum standard; P2 = The number of expert panel members that indicated during part 2 (of stage 2) that the element considered should be a minimum standard.

**Supplementary table 3. Specific accommodation criteria within the minimum standards for geriatric emergency care in Belgium**

| **Accommodation criteria for conventional EDs** | **P1** | **P2** |
| --- | --- | --- |
| Clear signage and way finding (e.g. colour contrast, large labelling of rooms...) | 15/19 | - |
| Non-slip floors | 14/19 | - |
| Efforts for noise reduction (e.g. silent alarms, closed rooms) | 14/19 | - |
| Handrails in sanitary facilities (toilet, shower) | 18/19 | - |
| Handrails in corridors | 18/19 | - |
| A wheelchair accessible toilet | 19/19 | - |
| A large-face analogue clock in each patient room | 14/19 | - |
| Opportunity for one visitor and the patient to sit beside the bed of the patient | 17/19 | - |
| Night-time lighting in the sanitary facilities | 16/19 | - |
| Natural light or dimmable lighting* | 18/19 | - |
| Raised toilet seats* | 16/19 | - |
| Calendar with day and date* | 15/19 | - |

e.g. = example given.

*This element is not a minimum standard for conventional EDs, but is for **EDs with geriatric-focused observation beds**.

P1 = The number of expert panel members that indicated during part 1 (of stage 2) that the element considered should be a minimum standard; P2 = The number of expert panel members that indicated during part 2 (of stage 2) that the element considered should be a minimum standard.

**Supplementary table 4. Article 3 of the Belgian federal legislation on the hospital-based care programme for geriatric patients**

| **The Belgian Royal Decree of the 29^th^ of January 2007 establishing the standards to be met by the care programme for geriatric patients (text updated on the 18^th^ of April 2014)** |
| --- |
| *Art. 3 the care programme for the geriatric patient addresses the population of geriatric patients, on average older than 75 years, who require a specific approach for several of the reasons listed below:*   1. *Fragility and limited homeostasis;* 2. *Active polypathology;* 3. *Atypical clinical presentation;* 4. *Disturbed pharmacokinetics;* 5. *Risk of functional deterioration;* 6. *Risk of deficient nutrition;* 7. *Tendency to inactivity and bedriddenness, with increased risk of institutionalisation and dependence in activities of daily living;* 8. *Psychosocial problems.* |
